# Microstructure Evolution in Dry‑Coated Sulfidic All‐Solid‐State Battery Cathodes: Investigating Core–Shell Particle Morphology

**DOI:** 10.1002/advs.78248

**Published:** 2026-10-12

**Authors:** Michael Wolf, Aaron Stoeckle, Rebecca Wilhelm, Maximilian A. Plass, Milad Hosseini, Johannes Kriegler, Korbinian Huber, Simon Lux

**Affiliations:** ^1^ Institute of Business Chemistry University of Münster, Leonardo Campus 1 Münster Germany; ^2^ Bayerische Motoren Werke AG München Germany; ^3^ TUM School of Natural Sciences Department of Chemistry and Catalysis Research Center Chair of Technical Electrochemistry Technical University of Munich Garching Germany; ^4^ Max Planck Institute for Solid State Research Stuttgart Germany; ^5^ Fraunhofer Research Institution for Battery Cell Production (FFB) Münster Germany

**Keywords:** interface, mechanofusion, microstructure, mixing, positive electrode, solid‐state electrolyte, solvent‐free

## Abstract

This study investigates continuous extruder mixing and high‑shear batch mixing to promote the formation of core–shell‐like particle coatings for enhanced interfacial contact in dry‑coated sulfidic all‑solid‑state battery cathodes containing 82 wt.% high‐Ni NCM85 (LiNi_0.85_Co_0.10_Mn_0.05_O_2_, particle size D_50_ 3.8 µm) cathode active material and 0.5 wt.% polytetrafluoroethylene (PTFE) binder. Particle morphology and electrode microstructure are analyzed by particle size measurements, electron microscopy, and X‐ray characterization. Morphological observations indicate that extrusion yields a robust PTFE fibril network and a more homogeneous electrode surface, where deformed Li_6_PS_5_Cl solid electrolyte particles suggest a core–shell‐like coating. Batch mixing resulted in reduced electrode quality, tensile strength, and microstructure homogeneity. Despite its dedicated mechanofusion process and higher specific mechanical energy input (∼ speed, time), a distinct core–shell‐like coating is not evident for such a small‐particle system. Charge transport features are analyzed in symmetric cells under blocking conditions. Batch‑mixed electrodes attain higher electronic conductivity, whereas extruder‑mixed electrodes achieve higher ionic conductivity. These differences likely arise from microstructural features. Electrochemical characterization of half‑cells demonstrates the enhanced performance of extruder‑mixed electrodes, including higher active material utilization, reduced overpotentials, and improved rate capability. Overall, extruder mixing provides a scalable and effective route for engineering the electrode microstructure.

## Graphical abstract

Mixing method directly governs interface engineering and electrode morphology during the dry coating of all‐solid‐state battery cathodes. While high‐shear batch mixing yields poor electrode quality and lacks core–shell‐like features, continuous extruder mixing enables strong binder fibrillation and a core–shell‐like coating between the active material and solid electrolyte. Consequently, continuous extrusion offers an efficient processing route for small‐particle systems, significantly enhancing charge transport and overall electrochemical performance.

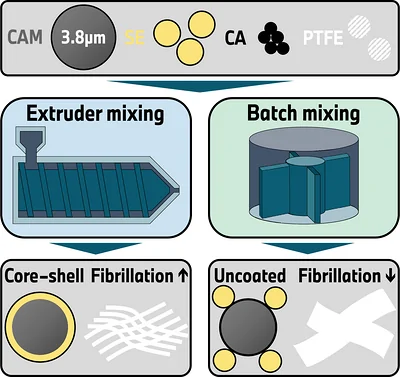

## 1 Introduction

The transportation sector is undergoing a rapid transition toward electrification, driven by the urgent need to reduce greenhouse gas emissions and mitigate climate change. Battery electric vehicles (BEVs) have great potential to decarbonize the transportation sector based on their emission‐free powertrain. However, the environmental impact of BEVs also depends on their carbon footprint during production. Especially, the state‐of‐the‐art manufacturing of lithium‐ion batteries (LIBs) involves high energy consumption and toxic solvents, which can substantially influence their environmental benefits [1]. The projected 40% BEV global market share in 2030 [2, 3]. highlights the importance of developing more efficient and sustainable battery cells.

While LIB‐based BEVs have initialized a global shift toward electric mobility, concerns about safety, energy density limits, and environmental impact give motivation to explore alternative battery technologies [4]. This drives the research focus toward next‐generation battery concepts that can overcome these limitations and offer improved performance and sustainability.

All‐solid‐state batteries (ASSB) have emerged as a strong candidate to address several of these challenges associated with conventional LIBs. ASSBs have the potential for higher energy density, improved safety, and enhanced thermal stability due to the use of non‐flammable solid electrolytes (SE) [4]. Among solid‐state chemistries, sulfidic SE are particularly promising because of their high ionic conductivities and favorable processing properties [5]. A key challenge in ASSBs is the formation of durable interfacial contacts between particles within electrodes. In ASSB cathodes (i.e., the positive electrodes), the interfaces among SE and cathode active material (CAM) particles govern ion transport and mechanical integrity upon cycling. Poor interfacial contact leads to high interfacial resistance, sluggish charge transfer, and degradation, which collectively degrades the battery performance and cycle life [5]. Unlike conventional liquid‐electrolyte systems, where particles are well connected through the liquid phase, the interfaces in solid‐state demand microstructure engineering based on optimized electrode design and processing [6, 7]. Consequently, achieving durable interfaces is critical for developing well‐performing ASSB cells. In fact, improved solid‐state interfaces can significantly reduce the external pressure on the cell stack required to maintain good interparticle contact and ionic conduction during battery operation [8]. Reducing the required pressure below 1 MPa allows simplified cell designs and packaging, making the batteries more practical for commercial applications such as BEVs [9].

Within this context, the role of mixing becomes decisive for advancing ASSB cathodes. On the one hand, achieving a uniform distribution of CAM, SE, carbon additive (CA), and binder ensures continuous ionic and electronic pathways throughout the electrode. Particle agglomerates and pores lead to bottlenecks in charge transport, increased interfacial impedance, isolated CAM, and particle cracking due to uneven distribution of mechanical stress during processing, which can ultimately compromise battery performance [1]. This can be prevented through the use of ductile, sulfidic SEs such as Li_6_PS_5_Cl (LPSCl) [10] with a low Young's Modulus of 10–30 GPa [11] that can plastically deform and cover hard CAM materials such as NCM with a high Young's Modulus of 100–200 GPa [12]. These coatings are often referred to as core–shell particles and can be induced by high shear forces using a tailored mixing technique called mechanofusion (MF) [13, 14, 15]. The deformability of LPSCl is mostly due to the presence of easily movable anionic groups in the crystal structure, which offer low resistance to shear deformation [16]. Elevated temperatures and thermal expansion make LPSCl even more ductile by increasing atomic spacing and reducing atomic bonding [17]. Core‐shell particles feature an increased active surface area between CAM and SE, minimize Li^+^ diffusion distances within CAM particles, and prevent contact loss during cycling [18]. An optimized core–shell structure can increasing CAM utilization by promoting ion percolation and access to active surfaces. Ultimately, cathodes with intrinsically robust interfaces are anticipated to improve rate capability, prolong cycle life, and support low‐pressure operation of ASSB cells [13, 18].

MF matches well with dry battery electrode (DBE) production based on polytetrafluoroethylene (PTFE) binder fibrillation – both requiring high shear forces during mixing [19]. Dry coating aims to eliminate the need for toxic solvents, drying, and solvent recovery, which significantly reduces energy consumption and CO_2_‐emissions during electrode production [1]. In addition, avoiding solvents prevents degradation of sulfidic SEs [1, 20, 21], thereby preserving their high ionic conductivity and improving battery performance.

The field of DBE manufacturing encompasses several solvent‐free concepts, including hot‐melt processing, brush application, electrostatic spraying, and calender‐based film formation. Among these, calendering offers the highest technology readiness level as well as most promising key performance indicators (KPIs) has been demonstrated on mass‐production‐relevant scales [22]. Before electrode formation through calendering, both Maxwell and DRYtraec calendering concepts use high‐shear powder mixing to initiate PTFE fibrillation. When compared to tumblers, mortar mills, and planetary mixers, both high‐shear mixing and extrusion/kneading techniques are efficient and highly scalable, despite requiring high capital expenditure (CapEx) [23]. The present study shall focus on the latter two, where mixing must achieve high PTFE fibrillation and MF to enable CAM–SE core‐shell coating. MF is typically performed using a high‐shear batch mixer [13, 14]. Continuous extrusion is an alternative mixing technique for dry coating. This method provides high shear forces, precise temperature control, and customizable screw designs to achieve the desired mixture homogenization and PTFE fibrillation [24]. In general, potential limitations for continuous mixing in ASSB DBE manufacturing may be the relatively short retention time, the need for a preceding premix step [25, 26], and an extra milling step afterward to break down coarse granules [27]. Both, extruder and high‐shear batch mixing are well scalable to match roll‐to‐roll calender‐based dry coating processes [28].

This study emphasizes the role of mixing and MF in dry coating sulfidic ASSB cathodes by investigating continuous extrusion and high‐shear batch mixing. Initial batch mixing trials were conducted to identify suitable conditions for MF and sufficient PTFE fibrillation to allow the fabrication of electrode films. The goal was to produce batch‐mixed electrodes with high homogeneity and mechanical stability and to achieve core–shell coatings between NCM (CAM) and LPSCl (SE). The microstructure, morphology, and chemical composition of composite powders and electrodes were characterized via scanning electron microscopy (SEM), energy dispersive X‐ray spectroscopy (EDX), X‐ray photoelectron spectroscopy (XPS), X‐ray diffraction (XRD), laser diffraction, and tensile tests. Moreover, the effects of the different mixing processes on the tensile strength and electronic and ionic conductivities of dry‐coated electrodes are compared. The electrochemical performance was further assessed through electrochemical impedance spectroscopy (EIS) and charge rate tests in cathode half‐cells with indium‐lithium alloy anodes. Additional chronoamperometry tests were conducted to further elucidate the rate capability and CAM utilization. Ultimately, this work correlates material properties, mixing conditions, and electrochemical performance to advance the development of dry‐coated cathodes for sulfidic ASSBs.

## 2 Results and Discussion

In the following, extrusion and batch mixing are compared at multiple levels, from parameter selection to material changes and electrode properties. Key considerations include (1) the selection of mixing parameters, (2) their effects on the CAM–SE interface, (3) SE alteration by mixing in dry room atmosphere, and (4) PTFE fibrillation. As PTFE fibrillation in dry‐coated sulfidic ASSB cathodes has been investigated in a previous study, suitable process conditions were adopted for this work [29]. Therefore, the following section focuses on the formation and microstructural characteristics of CAM–SE core–shell particles.

### 2.1 Comparison of Mixing Concepts and Powder Morphology

#### 2.1.1 Continuous Mixing

Continuous mixing was performed using a twin‐screw extruder. Practical processing parameters, including temperature and screw configuration, were derived from prior reports on extrusion‑based dry electrode manufacturing for LIBs [24, 27], and proved suitable for sulfidic ASSB cathodes. Due to the single feed inlet of the lab‑scale extruder, CAM, SE, CA, and PTFE binder were pre‑mixed in a planetary mixer that formed a coarse dispersion, which enabled continuous powder conveying through the extruder barrel without screw clogging. A schematic overview is provided in Figure 1a (bottom). The different screw elements are intended to facilitate specific process steps such as homogenization, fibrillation, and granule sizing [24, 30, 31, 32].

**FIGURE 1:**
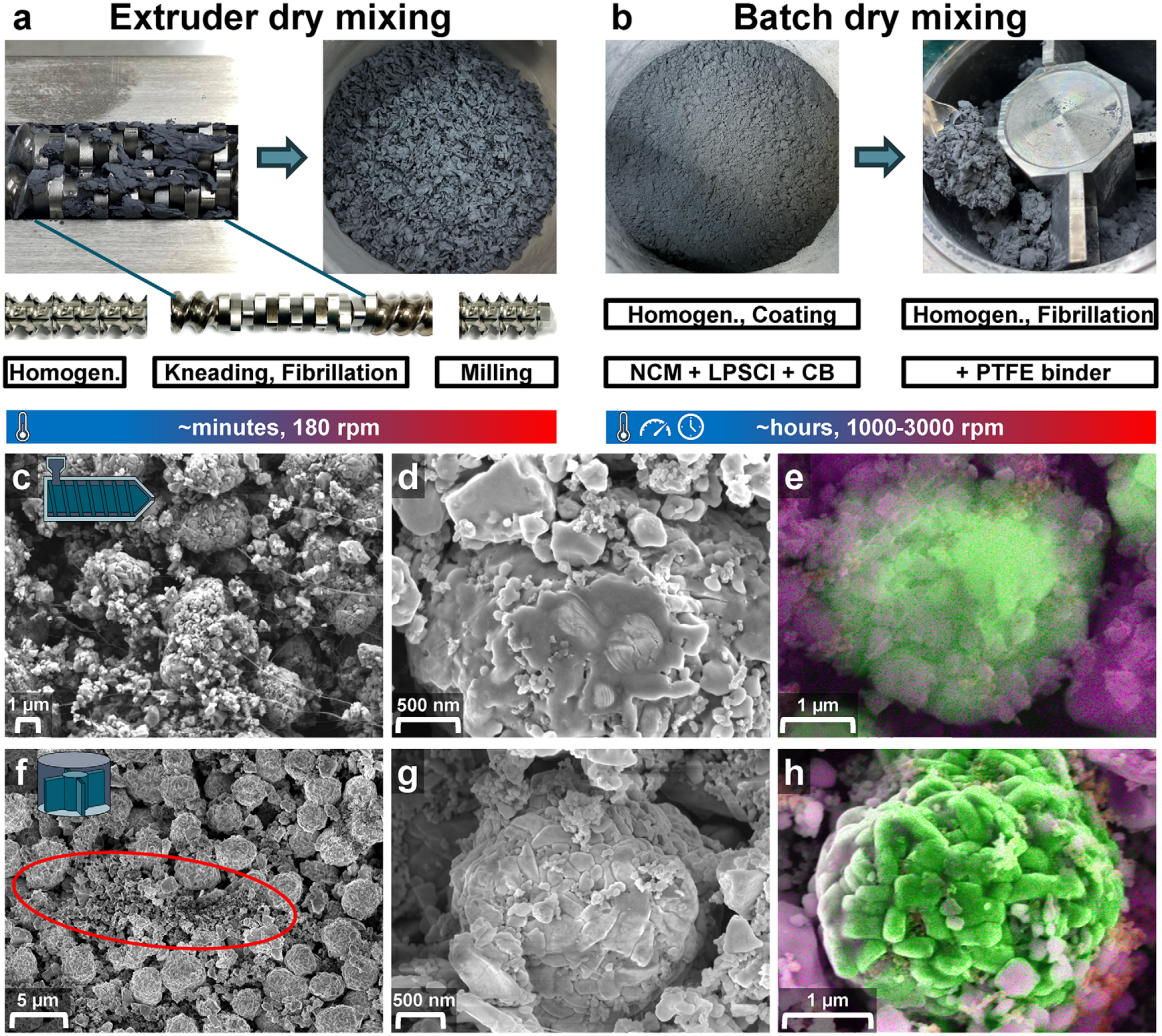
Continuous twin‐screw extruder‐mixing: transition of composite powder premix to fibrillated strings (left) and to fine granules (right) as end product, controlled by screw configuration and temperature (a). Batch‐ mixing: transition of composite powder after high‐shear mechanofusion (left) to fibrillated powder bulk (right) as end product, controlled by material sequencing, time, temperature, and rotational speed (b). Extruder mixing: SEM images of the composite microstructure with PTFE fibril network (c) and a close‐up view on deformed SE particles covering the CAM surface (d) with its corresponding EDX measurement (e). Batch mixing after PTFE fibrillation: SEM image of the composite microstructure with CA agglomerate highlighted by red ellipse (f). Batch mixing after MF step (without PTFE): SEM close‐up image of CAM surface (g) with its corresponding EDX measurement (h). EDX measurement are displayed by layered images combining the SEM image with O‐, Ni‐, Co‐, Mn‐Signals (green) referring to the CAM, and S‐, P‐, Cl‐Signals (purple) referring to the SE.

Extruder‐based mixing yielded a cohesive, fibrillated composite via a sequence of low‐ and high‐shear zones. Extruder mixing was initiated at room temperature in a low‐shear zone, where distributive flow elements promote powder homogenization without inducing premature PTFE fibrillation. In the subsequent fibrillation zone at 100°C, kneading elements imposed high shear on the powder, resulting in cohesive, fibrillated strings (Figure 1a top left). The milling zone yielded loose powder granules that were subsequently used for dry coating electrodes (Figure 1a top right).

Noteworthy, this mixing approach also addresses SE stability in dry room atmosphere. The enclosed extruder barrel and the relatively short residence time (≈ minutes) are intended to attenuate atmospheric exposure and reactions with residual moisture. Such reactions are accelerated at elevated temperature used for PTFE fibrillation [33]. The possible formation of Li_2_S, oxidic species, and other impurities is evaluated in Section 2.2.

#### 2.1.2 Batch Mixing

Batch mixing offers greater flexibility than extrusion in terms of sequential addition of materials, mixing time, and speed. A schematic overview is provided in Figure 1b (bottom). Practical parameters for batch mixing and MF coatings were selected based on initial trials (Figures S10 and S12) and on references [13, 14, 15]. Noteworthy, to evaluate potential competitive interactions between SE and CA during the MF process, the CAM coverage of SE and CA were tested isolated and when added simultaneously. SEM images in Figure S11 indicate a mixed coating of both SE and CA phases on the CAM surface rather than phase exclusion. This is consistent with recent findings by Kissel et al. [34] A MF mixing geometry was employed to generate high shear stress during the coating and fibrillation processes. The torque and power draw of the mixer were monitored to track changes in the powder. An increase in torque and power draw over time at constant speed can be interpreted as higher shear resistance of the material [35]. For a qualitative comparison, the maximum rate of change in power draw *ΔP Δt^−1^* was calculated for each mixing step (Table 1).

**TABLE 1:** Batch‐mixing routine detailing the sequence of material addition and process parameters.

Mixing step	Material	Speed [rpm]	Time [min]	Temperature [°C]	P_max_ [W]	ΔP Δt^−1^ [W min^−1^]	SME [kJ kg^−1^]
Homogenization	NCM, LPSCl, CB	1000	15	5	7	0.1	90
Coating		3000	45	5	98	2.3	5022
Homogenization	+ PTFE	1000	15	5	10	0.1	144
		2000	15	5	43	0.3	720
Fibrillation		2000	10	60	100	19.5	1164
**Total**			100				7140

Analogously to the extruder premix, batch mixing starts with the homogenization of CAM, SE, and CA at moderate speed (1000 rpm). To avoid SE agglomeration, the mixing chamber was cooled to 5°C [13, 14, 36]. Submicron particles such as SE and CA possess high surface areas, making them prone to agglomeration to reduce their surface energy [37]. With a low *ΔP Δt^−1^* of 0.1 W min^−^^1^, a quasi‐steady state in power draw was observed during the 15‐min homogenization step. Therefore, a constant shear behavior of the powder can be expected, indicating no microstructural changes.

The subsequent MF step was conducted at high rotational speed (3000 rpm) for 45 min to intensify shear stress between CAM and SE. Under sufficient shear stress, the ductile SE is expected to plastically deform and attach to the CAM surface. The power‐draw increased steadily with a *ΔP Δt^−1^* of 2.3 W min^−^^1^ for 10 min, then a plateau was reached. The increasing mechanical energy input to sustain the mixing speed indicates an increased shear resistance of the powder. Likely, the apparent resistance buildup may originate from rising particle–particle or particle–wall friction. Increased friction may result from additional deagglomeration (larger particle surface area) or cracked particles. In fact, a core–shell formation would reduce the fraction of free SE particles and increase the CAM particle size [14], thereby lowering the overall surface area of the composite and reduce friction. Thus, one can argue that a successful coating should enhance powder flow and reduce the shear resistance of the composite. After the MF step, a uniform, loose powder was observed (Figure 1b top left).

The coating step is followed by the addition and distribution of PTFE at moderate rotational speed (1000‐2000 rpm) to achieve a homogeneous binder distribution without triggering premature fibrillation. Over 15 min a consistent power‐draw with *ΔP Δt^−1^* 0.1‐0.3 W min^−1^ was observed, reflecting an unchanged powder morphology. To initiate PTFE fibrillation, the temperature was increased to the equipment maximum of 60°C. Then, a steep rise in power‐draw with a *ΔP Δt^−1^* of 19.5 W min^−^^1^ indicates an intensified shear resistance, associated to PTFE fibrillation with the powder becoming more cohesive. The powder granules as depicted in Figure 1b (top right) can be used for dry coating electrodes.

Noteworthy, the fibrillation time was limited by a maximum torque threshold of the mixer as the cohesion of the powder increases. Compared to the extruder, prolonged mixing times (≈ hours) can be beneficial for homogenization, coating, and fibrillation [37]. To prevent composite powder from entering the motor housing, the mixing chamber was continuously purged with pressurized dry air [37]. Consequently, in comparison with extrusion, the batch‐mixed powder was exposed longer to non‐inert atmosphere, thereby increasing the risk of SE degradation.

#### 2.1.3 Powder Morphology

Key aspects in dry powder mixing are the PTFE binder fibrillation and powder homogeneity. A cohesive fibril network is essential for the formation of dry‐coated electrode films via direct calendering. Moreover, a uniform distribution of SE and CA ensures ionic and electronic connection of the CAM toward the separator and current collector (CC).

Figure 1c depicts an SEM image of the microstructure of extruder powder granules. Fine PTFE fibrils are visible, interweaving and connecting larger CAM with smaller SE particles in all spatial directions. CAM, SE, and CA appear well distributed without visible agglomeration or CAM particle cracking (Figure S1a,b). In fact, CAM particles are covered by SE particles, indicating efficient mixing by extrusion.

Moreover, Figure 1d indicates an added layer on top of CAM particles, compared to the pristine material (Figure S2). Apparently, deformed and flattened SE particles firmly cover the CAM surface. EDX mapping of the extruded powder in Figure 1e depicts the distribution of characteristic signals of the CAM in green (Ni, Co, Mn, O) and of the SE in purple (S, P, Cl). A thin, uniform LPSCl coating can be observed that attenuates the EDX signal of the NCM particle underneath. This aligns with findings by Lee et al., who observed shear‐deformed LPSCl fractions covering the NCM surface [18].

Batch mixing yielded notably different outcomes. Although through batch mixing cohesive powder granules were formed, PTFE fibrils were scarcely observed (Figure 1f). This is indicative for insufficient fibrillation. In addition, several CA agglomerates were observed throughout the powder as depicted in Figure 1f. It is likely that CA agglomerates formed after PTFE was added. This effect was likely promoted by the intense material circulation and long contact time during batch mixing. Thus, although the CA can be homogeneously distributed at first as it was demonstrated by initial tests (Figure S10d–f), it likely was later incorporated into PTFE clusters. Notably, such PTFE clusters were not found with SE. Horst et al. employed a similar dry batch mixing process and initially observed well‐distributed CA adhering to the NCM surface [38]. After PTFE was added and mixed, it trapped the CA. Huber et al. and Damnali et al. reported a similar affinity between PTFE and CA [24, 27] Such clustering phenomena can compromise mechanical integrity of the electrode and hinder charge transport [39]. In contrast, the extruder mixes the powder in discrete segments and over a shorter residence time, which may prevent clustering more efficiently.

Furthermore, SEM images show that CAM particles after batch mixing (Figure 1g) appear similar to the pristine material (Figure S2). Deformed SE particles covering the CAM surface are not visible. The corresponding EDX mapping in Figure 1h reflects that the CAM particle surface is largely free of SE, indicating that no conformal SE layer was produced nor was the CAM surface morphology modified.

In initial tests (Figure S10), the applied MF process via batch mixing was successfully demonstrated with a larger polycrystalline NCM (D_50_ = 8.7 µm). A side‐by‐side SEM and EDX comparison of pristine and core–shell particles in Figure S4 indicate an added SE layer on top of this larger CAM. The main difference from the original polycrystalline NCM in Figure 1 is its smaller particle size (D_50_ = 3.8 µm). While small CAM particles are expected to experience higher particle–particle friction due to a higher surface area, larger particles transmit greater impact energy during collisions. Assuming spherical CAM particles of equal density, the particle mass and thus the kinetic energy (E_kinetic_ = ½ *m ν* ^2^) scales with particle size (E_kinetic_,_particle_ ∝ D^3^). Consequently, the large CAM (D_50_ = 8.7 µm) exhibits approximately 12‐fold higher kinetic energy than the small CAM (D_50_ = 3.8 µm). Frankenberg et al. described a similar force scaling effect for MF coating [37, 40]. Noteworthy, reports on MF typically used similarly large particles (D_50_ = 10–14 µm) [13, 14]. To achieve such a coating via batch mixing on smaller CAM particles, an increased mixing speed *ν* is intuitive (E_kinetic_,_particle_ ∝ *ν* ^2^). Since the specific mechanical energy (SME) input by extrusion (≈ 300 kJ kg^−^^1^) was substantially lower than that of batch mixing (≈ 7100 kJ kg^−^^1^) (Equations 2 and 3), the prevailing stress mode may be more relevant. Compared with predominantly impact forces in the batch mixer, the extruder likely imposes higher shear stress that more effectively deform such small SE particles. In general, smaller CAM particles are preferred in ASSB dry‐coated electrodes since they establish a higher contact area with the SE and are less prone to particle cracking in dry coating.

Despite these advantages of extruder mixing, industrial implementation requires further assessment. Since the used 11 mm extruder and throughput of 300 g h^−1^ allowed efficient process and product control, this may not be directly translatable to larger equipment, because screw geometry, residence‐time, SME input, and heat‐transfer conditions can change. The feasibility of slightly larger extruders for LIB dry coating has been demonstrated successfully [23]. Beyond homogenization, particle‐particle and particle‐wall friction significantly influence PTFE fibrillation and CAM–SE interfacial quality. While achieving target KPIs at scale may require advanced screw configurations or multi‐stage extruder layouts [41], these designs must be balanced against overall process efficiency, including premixing, heat transfer, and downstream processing. Furthermore, high frictional forces necessitate careful evaluation of mechanical wear and potential product contamination [23]. These factors highlight the need for pilot‐scale validation to comprehensively assess industrial viability and economics.

### 2.2 Microstructure Evolution of Powder Mixing

To better understand how batch mixing influences microscopic features of the composite, morphological properties of the powder are compared before and after the MF step (prior to PTFE addition). XRD measurements were conducted to probe deviations in phase composition and crystal structure. The XRD patterns of both samples are shown in Figure 2a,b. Both patterns appear nearly identical in terms of peak position, intensity, and width, which indicates consistency before and after batch mixing. The presence of well‐defined LPSCl diffraction peaks after mixing demonstrates that the SE retains its crystalline structure.

**FIGURE 2:**
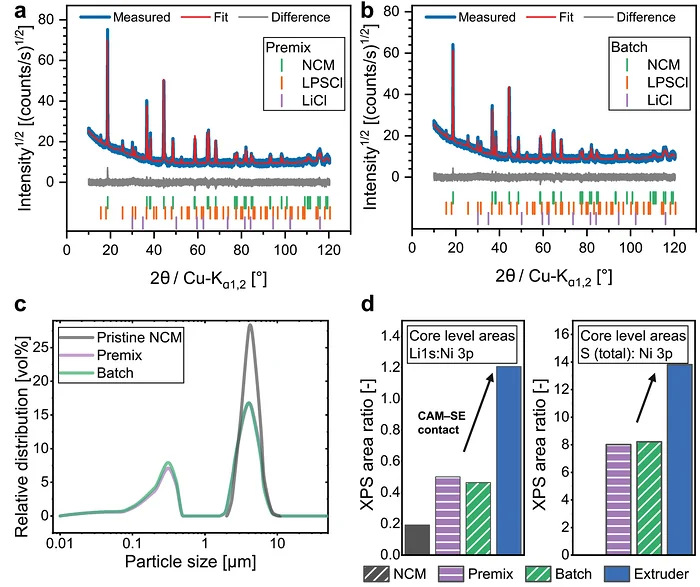
XRD patterns and results of Rietveld refinements of powder premix (a) and after batch mixing (b) (without PTFE). Laser diffraction measurements s howing relative (PSD in vol% of pristine NCM, powder premix, and after batch mixing (c). Based on GL(30) fits, areas of respective core levels are put into relation to indicate the degree of CAM surface coverage by SE species (d).

The influence of the mixing procedures on the microscopic properties of the composites have been investigated using quantitative Rietveld refinements. For this, the instrumental broadening has been determined with the use of a standard reference material beforehand, and all components of the composites have been measured and refined separately and in pure state. The phase contents are shown in Table S2. Phase contents of NCM, LPSCl, and amorphous phase are similar in both mixtures. Slight deviations might arise from inhomogeneities in the powder. Impurities of LiCl could hardly be observed in both composites due to its very low fraction [42].⁠ The mixing procedures do not seem to alter the microstructure or crystal structure of the NCM particles since no significant variation neither of the volume weighted mean column length (200–350 nm), nor of the microstrain (e_0_ ≈ 5 × 10^−4^) or the unit cell volume (+0.04 %) was observed. These observations indicate that batch mixing does not affect domain/crystallite size nor induces lattice strain in the CAM that might have been caused by the energy input and mechanical stress necessary for a core‐shell‐like coating [43]. In contrast, Hou et al. reported a relatively strong change after successful core‐shell coating of NCM, which was associated with macroscopic strain and structural disorder [44]. Overall, no chemical alteration of the NCM surface was detectable by XRD.

To corroborate these results, laser diffraction measurements were conducted to evaluate possible changes in particle size distribution (PSD) (without PTFE). Figure 2c shows the composite powder measurements before and after batch mixing, alongside the pristine NCM for comparison. All three samples show a primary peak at 3.8 µm, matching the D_50_ particle size of the pristine NCM. This indicates that neither particle fracture nor abrasion occurs. The reduced peak height observed for the premix and batch‑mixed powders results from a secondary peak at 0.3 µm associated with the SE. Both samples align well without major differences in height or shape of the primary and secondary peak. A more detailed comparison is provided in Table S2. In case of a successful core–shell coating, (1) the large fraction consisting of SE‐coated CAM particles would be expected to increase in size and volume fraction, and (2) the small fraction representing residual SE that is not coating the CAM particles to decrease, as observed by Kit et al. [14].

These results were complemented by XPS measurements that allow a more surface‐sensitive analysis of the composite morphology. For a semi‐quantitative analysis, GL(30) fits were added to the sulfur species and transition metals to extract the areas of the respective core levels, without adding further physical meaning. These XPS spectra in the low binding energy (BE) region can be found in Figure S5a. Two distinct Li 1s phases on the NCM surface can be identified. Intercalated lithium can be found at 54.0 eV and lithium surface species can be found at 55.2 eV [45]. Ni 3p can be found at 67.0 eV [46, 47]. In the S 2p spectrum, Li_2_SO_4_, LPSCl, and Li_2_S phases can be found at BEs of S 2p_3/2_ at 168.5, 161.5, and 160.3 eV, respectively [45, 47, 48]. Assuming a core–shell structure and if the outer shell (LPSCl) and the inner core (NCM) species are known, the attenuation of the core signal by the shell signal can be estimated. The core‐level area ratios can be interpreted as the ‘degree of coverage’ of the CAM–SE interface. Higher ratios indicate stronger attenuation of the CAM signal, reflecting an increased SE contribution and more extensive surface coverage. In the following, the pristine NCM, premixed, batch‐mixed, and extruder‐mixed powders are compared with respect to the CAM–SE contact. Figure 2d assesses two ratios: Li 1s (surface + LPSCl) / Ni 3p on the left and total S / Ni 3p on the right. Compared to the pristine NCM stoichiometry, all samples exhibit increased values. While the premix and batch‐mixed samples share similarly low values (Li/Ni 0.5; S/Ni 8.1), the extruder‐mixed sample shows increased Li/Ni and S/Ni ratios (1.2 and 13.8, respectively). Higher values indicate an improved CAM–SE interface. In contrast, a large CAM material reaches values of Li/Ni 4.4 and S/Ni 79.2, indicating a more consistent core–shell structure. Figure S5b compares the Ni 2p signal in the high BE region. For the NCM, two distinct peaks are clearly visible. Although slightly attenuated, both peaks remain visible for the premixed and batch‐mixed sample, indicating no complete coverage of the NCM phase. The extruder‐mixed sample shows both peaks largely disappeared, which reflects a well‐covered NCM surface. Kim et al. observed a similar reduction of the Ni 2p signal after MF coating NCM with LPSCl [13]. These findings are consistent with the results from SEM/EDX, XRD, and PSD, which show that the MF batch mixing process achieves insufficient SE coverage of the small particle size CAM. Instead, extruder mixing establishes enhanced interfacial contact between CAM and SE regardless of particle size. The theoretical shell thickness for the applied material system can be estimated based on the work of Kissel et al. (Figure S13) [34].

Additionally, XPS analysis was used to monitor possible SE phase losses during mixing in dry room atmosphere. Figure 3 depicts the S 2p spectra from SE composition changes of LPSCl. The LPSCl phase was found at a BE of 161.5 eV in all samples, with a FWHM of 1.1 eV (premix), 1.2 eV (batch), and 1.6 eV (extruder). Please note, that for simplicity all BEs and FWHM are only given for the S 2p_3/2_ peak [49]. An additional feature, corresponding to Li_2_S was observed toward a lower BE of 160.3 eV for the batch (FWHM = 1.1 eV) and extruder (FWHM = 1.2 eV) samples [45, 47]. In contrast to that, Li_2_SO_4_ was again observed for all samples at a BE of 168.5 eV, with a FWHM of 0.9 eV (premix), 1.0 eV (batch), and 2.1 eV (extruder) [49]. The sketch below the spectra displays the LPSCl phase purity, calculated from the corresponding peak area ratios. It has to be considered that XPS is a surface‐sensitive technique with a penetration depth of several nm and may not accurately reflect bulk properties of the material. The premix remains near 100 %, which is intuitive given the lack of exposure to dry room atmosphere and solvent. Despite longer mixing time and exposure to dry room atmosphere, batch mixing largely preserved the LPSCl phase (≈ 90%). Noteworthy, very small amounts of oxidized species were found, which aligns with the results of Kim et al. [13]. The extruder with a shorter residence time in dry room atmosphere exhibits a significant reduction in LPSCl phase (≈ 60%). The estimated layer thickness of contaminants on the LPSCl surface reached ≈ 1 nm for the extruder sample, while it was roughly one magnitude lower for the premix and batch‐mixed samples. A reasonable explanation could be the difference in temperature. Most of the batch mixing time was conducted under cooled conditions at 5°C, decelerating eventual reactions of LPSCl, while the extruder operated at 100°C. As described in our previous work, higher process temperatures are likely to accelerate degradation reactions of LPSCl [29].

**FIGURE 3:**
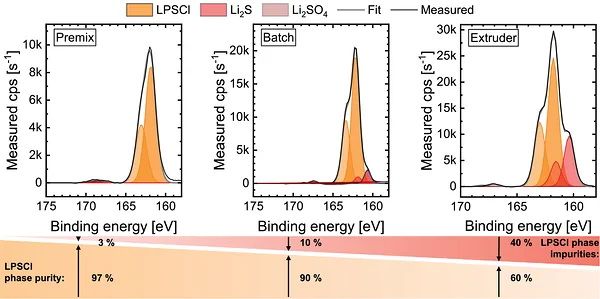
XPS S 2p 3/2 spectra referring to the LPSCl species at the binding energy 161.8 eV, Li_2_S at 160.3 eV, and Li_2_SO_4_ at 168.5 eV; surface‐charge corrected to adventitious carbon (284.8 eV) of the pristine NCM. LPSCl phase normalized to the total S phase.

The formation of these contaminants, predominantly Li_2_S, is likely initiated by H_2_O and CO_2_ from the atmosphere, and LiCl and Li_2_CO_3_ are the most prevalent degradation products. Mechanistically, LPSCl forms Li salts upon degradation as described by the reaction equations proposed by Chen et al. [50] and Naillou et al. [51]:

$$\begin{array}{ccc} & & Li_{6}PS_{5}\mathrm{Cl}+\left(1+4x\right)H_{2}O+CO_{2}\\ & & \;\to \;Li_{3}P{\left(S_{1-x}O_{x}\right)}_{4}+\;\mathrm{LiCl}+Li_{2}CO_{3}+\left(1+4x\right)H_{2}S↑ \end{array}$$

$$\begin{array}{c} Li_{6}PS_{5}\mathrm{Cl}+4H_{2}O\;\to \mathrm{LiCl}+Li_{2}S+Li_{3}PO_{4}+4H_{2}S\;↑ \end{array}$$

Therefore, the degree of LiCl and Li_2_CO_3_ formation would be also reflected in the phase purity in Figure 3. Such surface impurities and the reduction of LPSCl phase can lower the ionic conductivity of composite cathodes, which will be discussed in Section 2.4.

### 2.3 Electrode Manufacturing

The extruded and batch‐mixed powders were subsequently processed on a heated two‐roll calender to form electrode films. Both mixtures formed a cohesive film within a single asymmetric calendering step (unequal roll speeds) reaching the targeted average areal capacity of 3.2 mAh cm^−2^ (22.3 mg cm^−2^). The difference in roller speed is referred to as shear ratio and defined by Equation S1. Notably, both films show non‐uniform edges, likely due to the coarse granule size. However, additional powder milling was avoided to minimize dry room exposure.

Macroscopically, the extruder‐based electrode was free of cracks or voids, indicating a well fibrillated binder network (Figure 4a,c). Despite the homogeneous microstructure of the powder, alternating bright and dark surface regions were observed on the film. However, the SEM image in Figure 4c indicates a largely smooth and uniform electrode surface without visible irregularities. Like the extruder‐mixed powder, its electrode film demonstrates a vast PTFE fibril network (Figure S3a). The high‐shear calendering step seems to enhance fibril formation and uniaxial alignment.

**FIGURE 4:**
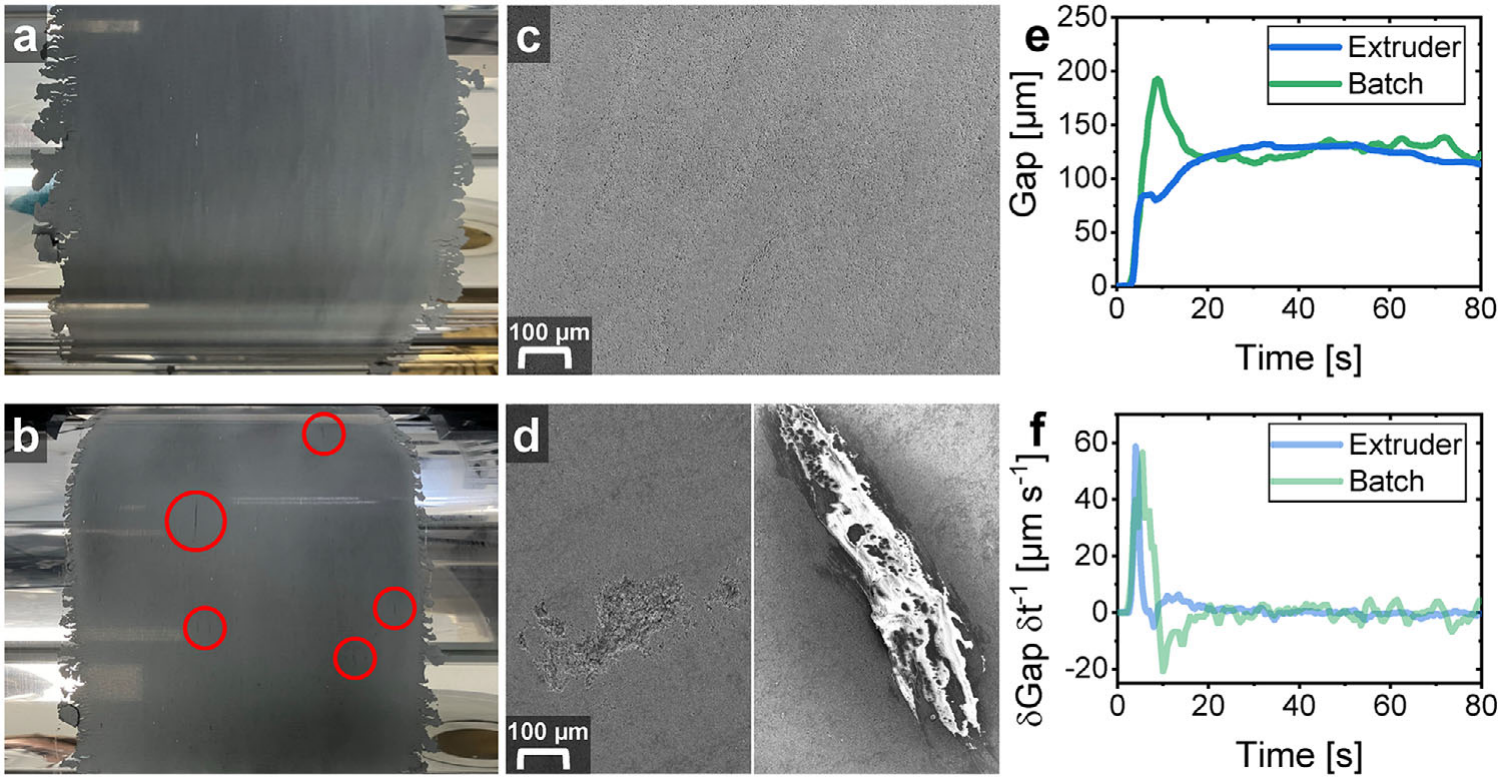
Dry‐coated electrode films adhering to the calender roller, based on extruder‐ (a, c) and batch‐mixed (b, d) powder, with the corresponding top‐view SEM image, respectively. Void defects at the surface are highlighted by red circles. Calender data during film formation: gap width recorded over time as an indicator for film thickness with a measuring frequency of 500 ms (e) and its time derivative δGap δt^−1^ (f). The applied calendering parameters are roller speed 0.1 m min^−1^, shear ratio 70 %, line load 100 N mm^−1^, and 60°C roller temperature.

The batch‐mixed film shows fewer alternating bright and dark surface regions. However, several longitudinal void defects are observable on the surface (Figure 4b). SEM images of the electrode surface in Figure 4d highlight surface defects and PTFE agglomerates. These likely stem from detached material sticking on the opposing roller, indicating reduced binding capabilities due to insufficient PTFE distribution/fibrillation. Notably, its electrode film exhibits more distinct PTFE fibrils than the batch‐mixed powder (Figure S3b).

Evaluating the powder‐to‐film calendering step provides additional information about the composite powder behavior. Monitoring the roller gap and torque serves as an indicator of the powder's shear behavior. Figure 4e compares the change in film thickness over time between the extruder‐ and batch‐mixed powders. After the onset at ∼15 s, both mixtures achieve a quasi‐steady state in calender gap width. Then, the gap variation for the extruder‐based electrode remains largely constant, whereas the batch‐mixed powder shows more fluctuations as visible by the 1st derivatives in Figure 4e. For the extruder‐based electrode, the change in thickness becomes very low and stable over time, indicating a favorable shear behavior of the powder. In contrast, the batch‑mixed composite exhibits larger fluctuations and does not attain a steady state, indicating non‐ideal shear behavior. The lower gap variance of the extruder‐based composite (6 µm) compared with the batch‐mixed composite (43 µm) may indicate differences in electrode film uniformity. Likewise, the average absolute value and variance of torque observed for the extruder‐based composite (21.8 Nm; 0.5 Nm) were lower compared to batch‐mixed (25.4 Nm; 3.0 Nm). This means the powders exhibit a different shear resistance and hence rotational force acting on the composite. The improved shear behavior of the extruded powder might be correlated with a more homogeneous electrode film and microstructure [35].

### 2.4 Electrode Microstructure

The electrodes produced by extruder and batch mixing were further characterized to correlate microstructural features with mechanical stability and electrochemical behavior.

A comparison in tensile strength of both electrodes is presented in Figure 5a,b. By pulling apart electrode die‐cuts, the tensile strength at the CC–coating interface and within the coating itself can be evaluated. Both electrodes experience a cohesive failure within the coating. In fact, the extruder‐based electrode with 29.4 N cm^−2^ shows a threefold higher cohesive strength compared to the batch‐mixed electrode with 9.3 N cm^−2^. That finding further corroborates the proposed poor PTFE fibrillation during batch mixing, as fewer fibrils reduce the mechanical cohesion among particles within the composite. Compared to the cohesive strength, the adhesive strength of both electrode configurations is higher and seems sufficient for their handling in downstream processes, i.e., cell assembly. Notably, the extruder‐based electrode reaches a mechanical stability comparable to state‐of‐the‐art LIB electrodes with literature values of 18.7 and 41.3 N cm^−2^ for dry‐ and wet‐coated samples [52].

**FIGURE 5:**
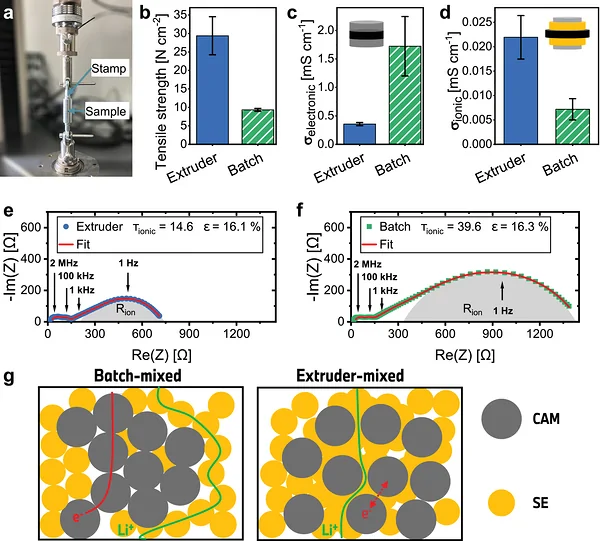
Tensile strength test setup (a), tensile measurement of electrodes (b), electronic conductivity measurement at 24 MPa, 25°C (c), and ionic conductivity measurement at 12 MPa, 25°C (d). Exemplary EIS spectra of extruder‐mixed (e) and batch‐mixed (f) electrode films with corresponding thicknesses of 80 / 73 µm and weights of 51.7 / 45.7 mg, respectively. Data points are displayed as markers and TLM fits as red line. Additionally, values of porosity ε and electrochemical tortuosity τ_ionic_ are provided. Scheme of microstructural electrode features of batch‐ and extruder‐mixed cathodes (g). Included error bars show standard deviation values.

To correlate the electrode microstructure with charge transport properties, electronic and ionic conductivities were measured. As shown in Figure 5c, the batch‐mixed electrode with 1.72 mS cm^−1^ exhibits a fivefold higher electronic conductivity than the extruder‐based configuration with 0.35 mS cm^−1^. The magnitudes of observed conductivities are consistent with literature [53]. This substantial discrepancy cannot be attributed to the nearly identical average porosity of free‐standing electrode films deviating by 0.2 % (Table S3). Moreover, the stronger CA agglomeration observed in the batch‐mixed composite (Figure 1f) may actually reduce the contribution of its CA phase to the electronic conductivity of the electrode. Another cause for the difference in electronic conductivity likely results from the core–shell‐like morphology observed for the extruder‐mixed composite. Because the SE shell‐like structure acts electronically insulating, its coverage of the CAM particles can reduce the contribution of the CAM phase to the electronic conductivity of the electrode, as described by Minnmann et al. [54] This effect may be particularly pronounced for high‐Ni NCM (≥ 80 mol% Ni), which exhibits an electronic conductivity on the order of 10^0^ mS cm^−1^ [55, 56], and for electrodes with a high CAM fraction of 82.0 wt.%. In contrast, CAM particles without an SE shell‐like structure, as observed in the batch‐mixed composite, can contribute more effectively to the electrode's electronic conductivity. These results align with the MF experiments conducted by Kim et al. [14].

The ionic conductivity was extracted from EIS measurements of symmetric cells with free‐standing cathode films in blocking conditions using a transmission line model (TLM) fit. With porosity considered, the actual volume fraction of the SE phase in the composite was determined (Equation S3). Both electrode configurations, extruder‐ and batch‐mixed, have comparable porosity of roughly 16 % and an effective SE volume fractions of 25.8 vol%, enabling a fair comparison (Table S3). As shown in Figure 5d, the extruder‐based electrode with 0.022 mS cm^−1^ exhibits a more than three times higher ionic conductivity than the batch‐mixed electrode with 0.007 mS cm^−1^. Related studies investigating LPSCl‐based cathodes with comparable compositions (≈ 80 wt.% CAM) achieve similar values in the range of ≈10^−2^ mS cm^−1^ [14, 39, 57, 58]. The difference in impedance of the symmetric cathode cells is highlighted by the Nyquist plots in Figure 5e,f. The impedance data was fitted according to the TLM model in Figure S7a. At high frequencies (> 3 MHz), the purely resistive SE bulk resistance R_bulk_ can be observed. The first two semicircles in the high‐ and mid‐frequency range are contributions from the separator R_Sep_ (SE grain boundaries, polymer binder) at ≈ 2 MHz and the anode interface R_InLi/Sep_ at ≈ 100 kHz, respectively, which are nearly identical for both electrode configurations. A third semicircle appears at low‐frequencies < 100 Hz that is characteristic of finite diffusion with a transmissive (or permeable) boundary and can be attributed to ion diffusion through the cathode [59]. Assuming non‐faradaic behavior (no intercalation) and due to blocking conditions, the obtained resistance R_ion_ can be attributed to mass transport in the ion‐conducting SE phase within the cathode microstructure. Notably, the thicker the electrode film, the longer the ions take to exit the finite diffusion length, i.e., lower frequencies [59]. Therefore, similarly thick and porous electrode samples were chosen for comparison. R_ion_ is significantly increased for the batch‐mixed electrodes. Consequently, extruder‑mixed electrodes facilitate more effective ionic percolation.

The negative effect of high CAM–SE coverage on electronic conductivity described before might be beneficial for ionic conductivity. In the extruder‐mixed electrode, CAM particles are well‐covered by a SE coating, whereas in the batch‐mixed electrode they remain largely uncovered. When the SE covers CAM particles and CAM particles are disconnected, the ion percolation network is likely more uniform with fewer interruptions (Figure 5g). Consequently, the extruder‑mixed electrode exhibits a lower (electrochemical) tortuosity of 14.6 than the batch‑mixed electrode with 39.6, as determined by Equation S7. Compared to literature, it has to be differentiated between simulation, geometric, and electrochemical methods. For LPSCl based electrodes with roughly 80 wt.% CAM and similar measurement methods, electrochemical tortuosity values were found to range from 10 [60] to 100 [53], dependent on processing and particle system. Accordingly, extruder mixing fosters favorable ionic transport in cathodes.

An additional contributing factor may be the correlation between powder handling in dry room atmosphere and LPSCl degradation. According to the surface composition obtained by XPS (Figure 3), the extruder‑mixed sample shows the most pronounced LPSCl degradation. The bulk conductivity of the SE material may be largely preserved. Nevertheless, the effective ionic conductivity of cathodes fabricated by extruder mixing is higher than in the batch‐mixed electrode. This leads to the assumption that the microstructural contribution can outweigh LPSCl degradation in composite cathodes. Increased homogeneity and interfacial contact between CAM and SE are considered to significantly enhance the ion percolation network in the extruder‐mixed configuration. Notably, bulk conductivities do not reflect the electronic and ionic connection of the CAM phase, that influences charge‑transfer resistance and CAM utilization, which will be discussed in the next section.

While cross‐sectional imaging and 3D‐microstructure modeling was unavailable within the framework of the current work, the conclusions regarding microstructural differences of extruder‐ and batch‐mixed electrodes relay on a complementary correlation of bulk transport behavior, porosity, and surface morphology.

### 2.5 Cycling Performance of Cathode Half‐Cells

This section evaluates how mixing and different electrode microstructures affect the electrochemical performance of ASSB cells. The final laminated extruder‑ and batch‑mixed cathodes were assembled into single‑layer coin‑pouch cells with InLi anodes. Their main characteristics are summarized in Table 2. Independent from the mixing technique, all cells reach initial coulombic efficiencies (ICE) well above 80 %, which is typical for LPSCl‐based cathode half‐cells coupled with InLi anodes [39, 53, 61, 62, 63]. In the first cycle, the cells containing extruder‐based cathodes achieved charge capacities of 204 mAh g^−1^_NCM_, which is 9 % higher compared to the batch‐mixed configuration with 188 mAh g^−1^_NCM_. The high initial charge capacity of the former indicates increased accessibility of lithium from the CAM. CAM utilization refers to the CAM fraction that is ionically and electronically well connected. The enhanced CAM utilization observed for cells with extruder‐mixed cathodes possibly originates from fewer isolated CAM particles that are poorly connected and more difficult to electrochemically access at 0.1 C (Figure 5g) [53, 64]. Likewise, the enhanced CAM–SE interface in the extruder‑mixed cathode can prevent contact loss during charging, which arises from the contraction of high‑Ni NCM particles upon delithiation within the applied voltage window [65]. Thus, CAM utilization depends not only on the cathode composition but also on the mixing process.

**TABLE 2:** Characteristics of laminated electrodes used for cathode half‐cells (cold, uniaxially compacted at 800 MPa).

Electrode	Density [g cm^−3^]	Porosity [%]	Areal loading [mg cm^−2^]	Areal capacity [mAh cm^−2^]	Coulombic efficiency [%]	1^st^ charge capacity [mAh g^−1^_NCM_]
Extruder	3.23 ± 0.11	12.6 ± 3.0	22.58 ± 0.62	3.26 ± 0.09	85.46 ± 0.22	204.09 ± 0.98
Batch	3.30 ± 0.09	10.8 ± 2.4	24.93 ± 0.47	3.58 ± 0.07	83.12 ± 1.17	187.50 ± 2.02

Voltage profiles of the first formation cycle of both cell configurations are depicted in Figure 6a. The cell with a batch‑mixed cathode shows a larger hysteresis along the entire voltage window (distance between charge and discharge curves at equal state of charge), indicating higher resistance compared to the extruder‑mixed configuration. At the onset of the charge curves (highlighted by red square), the batch‑mixed cell configuration shows an 0.05 V higher vertical voltage increase, characteristic of kinetic limitations on the cathode side [66]. Similar conclusions can be made from the differential capacity curves (dQ/dV) in Figure 6b. Three phase transitions characteristic of Ni‐rich NCM are observed [55]. The initial lithiated hexagonal phase (H1) of the layered crystal lattice starts transitioning from 3.6 V to monoclinic (M) until 3.8 V vs. Li^+^/Li [67]. As delithiation proceeds, the structure returns to a hexagonal phase (H2) at 3.9–4.1 V vs. Li^+^/Li. Subsequently, the high‐voltage hexagonal transition (H3) occurs at 4.2 V vs. Li^+^/Li. For the H1–M transition, two effects are visible. The extruder‑mixed cell configuration shows an earlier onset of the first anodic (charge) peak with high intensity, followed by a second peak of lower intensity. The cell with batch‑mixed cathode exhibits the opposite behavior, with a later onset and a more intense second peak, indicative of higher overpotentials and slower kinetics. The postponed first peak is described as ‘kinetic hindrance’. Slow kinetics are possibly caused by sluggish charge transport though the cathode or across the CAM interface. As both electrodes demonstrated higher electronic than ionic conductivities (Figure 5c,d), ionic limitations can be assumed [54].

**FIGURE 6:**
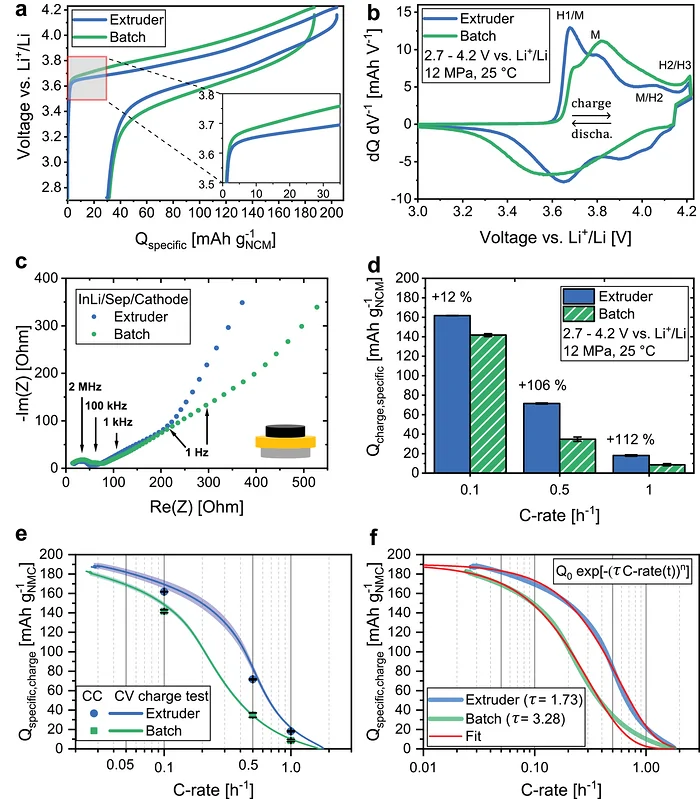
Cathode half‐cell formation data at 0.1 C: cell voltage profiles (a), differential capacity analysis (dQdV) graphs (b), and resistance contributions derived from EIS, including DRT peak fits (c) of first cycle, comparing extruder‐ and batch‐mixed electrodes. Corresponding specific charge capacities at different C‐rates during galvanostatic rate tests (d) and potentiostatic rate charge tests (e), including fit function with characteristic time constant τ (f). Included error bars / band show standard deviation values.

Furthermore, EIS measurements were conducted to better understand individual electrochemical processes. The Nyquist plots of both cell configurations are shown in Figure 6c. Analogously to the symmetric cathode cell, similar high and mid frequency contributions are observed (R_bulk_, R_Sep_, R_InLi/Sep_). At low frequencies < 1 kHz, the spectrum turns into a straight line. In general, the batch‐mixed configuration shows increased cell impedance, which agrees with the elevated overpotentials observed in Figure 6a. Notably, the straight upward line at low frequencies might be characteristic of finite diffusion in the cathode with a reflective (or non‐permeable) boundary, different to the low frequency semicircle observed for the symmetric cathode cell with a transmissive (or permeable) boundary [59].

Due to the complexity of the spectrum, a robust equivalent circuit model was not feasible. Instead, the distribution of relaxation times (DRT) was analyzed, based on a mathematical model describing an infinite number of RC elements with certain time constants τ* [68, 69]. Electrochemical processes can occur at the same time but typically at different time domains / frequencies. In DRT plots, each peak can be assigned to a process and the peak area quantifies its impedance. Details of the peak assignment are provided in the Supporting Information (Figure S7). It was found that the reduced cell impedance observed for the extruder‐mixed configuration may originate from a lower charge‐transfer resistance resulting from the enhanced CAM–SE interfacial contact. Related studies with similar cell setups attributed this mid‐to‐low frequency region at ≈ 1 kHz to the charge transfer process in the cathode [6, 8, 70]. Zhou et al. found that smaller SE particles improve CAM–SE contact and reduce charge‑transfer resistance, with the effect most pronounced at low stack pressure of 2 MPa [8]. However, the charge transfer resistance (P3 peak) is difficult to quantify and appears to be significantly smaller than the diffusion resistance (P4 peak). Thus, despite charge transfer and ion diffusion act in superposition on the cell kinetics, it is likely that primarily the electrode's ionic conductivity determine cell performance.

In the next step, the rate capability of the cathode half‐cells was assessed. The attainable capacity at high C‐rates is a key indicator of power density in ASSB cells. Galvanostatic charge rate tests were conducted because at high discharge currents InLi anodes tend to form In‐rich ‘barriers’ that impede ion transport and accessible capacity [71].

Figure 6d shows the results of specific capacities at different charge rates. Both cell configurations exhibit decreasing specific capacities with increasing C‑rate. Regardless of the rate, the extruder‐mixed cell configuration outperforms the cells with batch‐mixed electrodes. At 0.1C, moderate differences were observed, with the extruder‐mixed electrode delivering 162 mAh g^−1^_NCM_ versus 142 mAh g^−1^_NCM_ for the batch‐mixed electrode. At 0.5C, 72 mAh g^−1^_NCM_ and 35 mAh g^−1^_NCM_ were achieved by the cells with extruder‐ and batch‐mixed electrodes, respectively, and 18 mAh g^−1^_NCM_ and 9 mAh g^−1^_NCM_ at 1C. In fact, the extent of capacity decline also differs. Compared with 0.1 C, the extruder‐mixed cell configuration still achieves 44% at 0.5 C and 11% at 1 C, whereas the batch‐mixed configuration achieves only 25% and 6%, respectively. This trend highlights the improved rate capability of the extruder‐mixed cathode. Especially at higher rates where mass transport becomes limiting, its improved ionic conductivity is advantageous. Also, effects at the CAM–SE interface may be more pronounced at elevated rates [18]. Under high‑shear extruder mixing, deformation of the SE likely creates a larger and firmer contact area with the CAM, than the particle‑to‑particle point contacts formed during batch mixing. A comparable relationship was reported by Schlautmann et al. and Zhou et al., who showed that smaller SE particles increase CAM contact area and enhanced cell performance [8, 61]. Notably, the poor rate capability of both cell configurations may also be linked to the separator–anode interface resistance associated with their moderate fabrication parameters (0.5 t, 1 min) [71].

The results were further validated by chronoamperometry experiments that accelerate and deepen the cell evaluation by assessing rate capability across many C‐rates in a single potentiostatic cycle [72]. The typical exponential‐like current decay observed in Figure S9 was converted to specific capacities and C‐rates as shown in Figure 6e according to Equations 4 and 5. The chronoamperometry curves align well with the standard galvanostatic (CC) rate test results from Figure 6d. Due to possible InLi anode limitations, data at higher rates cannot be obtained. Rates below 0.05 C further minimize kinetic limitations compared to 0.1 C, allowing a more accurate assessment of the CAM utilization. At the minimum measurable rate of 0.03 C, the extruder‐mixed cell configuration reached a 5 % higher capacity with 188 mAh g^−1^_NCM_ versus the batch‐mixed configuration with 179 mAh g^−1^_NCM_. This is slightly less than the 9 % observed at 0.1 C, yet consistently the extruder‐mixed cell configuration appears to achieve higher CAM utilization.

The chronoamperometry data can be fitted with a mathematical function based on the C‐rate model proposed by Tian et al. and Wong et al. [73, 74]. Derived fit parameters can then be compared and electrode limitations identified. Figure 6f shows specific charge capacity vs. C‐rate of the measured chronoamperometry curve with the corresponding fit. Data fitting was performed with the fit parameters *Q_0_*, τ, and *n*. *Q_0_* describes the maximum attainable specific capacity of the CAM at the minimum rate (*C‐rate(t)* → 0). Proper fitting results were obtained using a specific capacity of 190 mAh g^−1^_NCM_, which is reasonable for a high‐Ni NCM within the voltage window of 2.7‐4.2 V vs. Li^+^/Li [75]. When reaching 0.01 C the fits of both cell configurations converge close to 190 mAh g^−1^_NCM_ (→ *Q_0_*). Both configurations appear to utilize a similar fraction of CAM. Yet their lower capacities at 0.1C emphasize pronounced kinetic limitations. Improving electronic and ionic connectivity of the CAM phase could increase the accessible active fraction at higher rates.

Since rates below 0.1 C are not practical for automotive applications, the specific charge capacities of *Q_0_* and 0.1 C are compared to estimate an effective CAM utilization θ*_effective_*:

(1) $$\theta_{effective}=\frac{Q_{0.1C}}{Q_{0}\;}\;\;\;\;\;\left[-\right]$$

An ideal value would be θ*_effective_* = 1. The effective CAM utilizations of the cells with extruder‐ and batch‐mixed cathodes are θ*_effective_* = 0.85 and θ*_effective_* = 0.75, respectively.

Moreover, the onset of the inflection point between low and high rate regions is reflected by the characteristic time parameter τ and can be correlated with rate capability [74]. In general, a smaller τ corresponds to a slower capacity decay of the curve and, consequently, improved rate performance of the cell. This aligns well with the different curve behavior in Figure 6f, where the batch‐mixed cell configuration with τ = 3.28 h declines earlier compared to the extruder‐mixed configuration with τ = 1.73 h. That agrees with earlier improvements observed and proposed for the extruder‐mixed cell configuration. Especially improved charge‐transport and transfer characteristics are associated with smaller values of τ [72, 73].

The stretching exponent *n* reflects the slope of the exponential decay and describes the rate‐limiting mechanism according to Yanev et al. [72]. Both cell configurations show values near 1.3, indicating similar limitation mechanisms. However, literature typically reports values for *n* ≤ 1 [72, 73, 74]. Notably, values greater than 1 may reflect resistance contributions from the InLi anode overlapping the cathode behavior.

In conclusion, ionic conductivity is likely the primary bottleneck for CAM utilization and rate capability. The formation data and the galvanostatic and potentiostatic rate capability tests demonstrate that extruder‐mixing is promising for enhanced ASSB cell performance.

## 3 Conclusions

This study provides a systematic comparison of continuous extrusion mixing and high‑shear batch mixing for producing dry‑coated sulfidic ASSB cathodes. The experiments elucidate powder mixing behavior, electrode fabrication, composite microstructure, charge‑transport, and electrochemical performance to establish material–process–performance relationships.

Surface analysis indicated that extruder mixing yields a highly fibrillated PTFE network and a more homogeneous distribution of CAM, SE, and CA on the composite powder Further morphological characterization suggests that SE particles are in close contact with the CAM surface, indicative of a core–shell‐like structure. Batch mixing indicated reduced PTFE fibrillation, increased carbon‐binder agglomerates, and did not yield evidence of distinct core‐shell‐like coating formation for the small‐particle CAM (D_50_ 3.8 µm).

SEM, EDX, XRD, laser diffraction, and XPS analysis revealed negligible surface modification of the CAM through MF batch mixing. Estimations of the apparent surface coverage of CAM with SE indicated an enhanced CAM–SE interface for the extruder‐mixed composite.

During electrode fabrication, the extruded powder formed defect‐free films with improved thickness uniformity and tensile strength. Batch‑mixed powders resulted in films with surface‐defects and reduced cohesive strength, correlating with insufficient PTFE fibrillation. Symmetric cells with ion‑ and electron‑blocking configurations were utilized to assess the charge‑transport behavior of the composite cathodes. Batch‑mixed electrodes exhibited higher electronic conductivity compared to extruder‐mixed, while the opposite was observed for the ionic conductivity. Monitoring the LPSCl phase purity by XPS indicated that the differences in ionic conductivity cannot be associated with SE degradation but instead depends on microstructure features. The apparent core‐shell‐like morphology suggested for the extruder‐mixed samples does not benefit the electrode's electronic conductivity, but it appears to prevent agglomeration and enhance ionic percolation.

Electrochemical analyses of cathode half‐cells with InLi alloy anodes further emphasized the advantages of extrusion. Cells with extruder‐mixed cathodes showed higher first‑cycle capacities, reduced overpotentials, and lower impedance contributions. Charge rate capability evaluated by galvanostatic and chronoamperometric tests was consistently superior for extruder‑mixed electrodes across all C‑rates. Based on chronoamperometry (i.e. potentiostatic cycling) experiments, an effective CAM utilization was derived, indicating that more CAM can be electrochemically accessed in the extruder‐mixed cathode. Evaluating the long‐term cycling stability of ASSB cells with these electrode designs remains the subject of a follow‐up study.

Overall, the results demonstrate that achieving sufficient electrode homogeneity, PTFE fibrillation, and CAM–SE contact via high‐shear batch mixing can be challenging for small particle systems in sulfidic ASSB cathodes. In this baseline comparison, where both mixing techniques are investigated individually as alternative processes, continuous extrusion provided a robust and scalable route to engineer these microstructural features, translating into enhanced mechanical integrity, ionic conductivity, and ASSB cell capabilities. Integrating both methods into a complementary hybrid mixing concept represents a promising pathway for further process optimization.

## 4 Experimental

All materials (except PTFE and LPSCl) were vacuum dried at 60°C for at least 12 h using an antechamber oven (MB‐VOH‐150, MBraun, Germany). Dry mixing and coating were conducted in dry room atmosphere < −40°C dew point. The slurry coating of the separator and the cell assembly were performed under inert gas atmosphere in Ar filled gloveboxes (H2O < 0.1 ppm, O2 < 0.1 ppm, MBraun, Germany).

### 4.1 Materials

The cathodes consist of poly‐crystalline LiNi_0.85_Mn_0.05_Co_0.10_O_2_ (NCM) (D_50_ = 3.8 µm, BASF, Germany) as CAM with an assumed reversible capacity of 175 mAh g^−1^_NCM_ and LPSCl (MITSUI MINING & SMELTING CO., LTD., JAPAN) as SE. Carbon black super C65 (Imerys, France) was used as CA, PTFE (Chemours, USA) as binder, and primed aluminum (ARMOR, France) as CC. The large CAM NCM has a particle size of D_50_ = 8.7 µm. The wet‐coated separator consists of the polyisobutylene (PIB) (Oppanol N 100, BASF, Germany) binder and LPSCl SE (D_50_ = 5 µm, (POSCO Jeong Kwan Solid Solution, South Korea). Xylene (Merck Millipore, Germany) and IBIB (Sigma Aldrich) were dried via molecular sieves (Merck Millipore, Germany) for at least 48 h. The coated sheets were vacuum dried at 60°C for 24 h and stored in the glovebox. Lithium‐indium alloy anodes were manufactured by pressing lithium foil (0.50 mm, 99.9 %, Albemarle, USA) and indium foil (0.75 mm, 99.99 %, Thermo Fisher Scientific, USA) according to an established method [76].

### 4.2 Mixing and Coating

#### 4.2.1 Dry Coating Cathodes

The dry‐coated cathodes contain 82.0 wt.% NCM, 15.5 wt.% LPSCl, 2 wt.% C65 and 0.5 wt.% PTFE binder. NCM, LPSCl, C65, and PTFE were homogenized in a planetary mixer (ARV‐310P, THINKY, USA) to obtain a powder premix.

A parallel twin‐screw extruder (Process 11, Thermo Scientific, Germany) at a constant feed rate of *ṁ* 0.3 kg h^−1^, a rotational speed of approximately *n* 180 rpm, and an effective torque of both shafts of approximately *M* 1.2 Nm (*M_0_* neutral engine torque) was utilized for continuous mixing. The screws measure 11 mm in diameter and were dried at 60°C for 24 h prior to use. The specific mechanical energy SME input by twin‐screw extrusion was calculated by the following Equation (2) [77]:

(2) $$SME=\frac{2\;\pi \;n\;(M\;-\;M_{0})}{\dot{m}}\;\;\;\;\left[kJkg^{-1}\right]$$

A high‐shear batch mixer (Picoline Nobilta, Hosokawa Alpine AG, Germany) with a temperature control unit, which was cooled and heated to 5°C and 60°C, respectively (equipment limit), was utilized for batch mixing. The mixing chamber was filled with *m* 50 g of composite powder, corresponding to roughly 50% of its total volume. The *SME* input by high‐shear batch mixing was calculated for each step with the corresponding time *t* and powder *P* (*P_0_* neutral engine powder) by the following Equation (3):

(3) $$SME=\frac{(P\;-\;P_{0})t}{m}\;\;\;\;\left[kJkg^{-1}\right]$$

The obtained powders were processed in a calender with two horizontally aligned heated rollers (GK‐300L, Saueressig, Germany) in constant force mode. In the first run, a coating width of 100 mm, a roller speed of 0.1 m min^−1^, a shear ratio of 70% (asymmetric roller speeds), a line load of 100 N mm^−1^, and a temperature of 60°C were applied for film formation via direct calendering (adhesion to roller). In the second run, the film is laminated onto the CC with a reduced LL of 50 N mm^−1^ and shear ratio of 0% (symmetric roller speeds). Al foil without primer was used to obtain freestanding films for ionic conductivity measurements.

#### 4.2.2 Slurry‐Coated Separator

The wet‐coated separator contains 95 wt.% LPSCl and 5 wt.% PIB binder. Its production followed the same manufacturing steps as described in our previous work [29].

### 4.3 Characterization

#### 4.3.1 Electronic Conductivity Measurement

Symmetric ion‐blocking steel/cathode/steel cells were assembled to determine the electronic resistance R_el_. 12 mm cathodes were compacted as described in 4.4.1. The cathodes were pressurized at 24 MPa in a PEEK cylinder in between steel pistons (CompreDrive, rhd Instruments, Germany). For the direct current (DC) polarization technique, voltages of 25, 35, 45, 55, and 65 mV were applied for 5 min each. Flat current plateaus at each potential were observed which are characteristic for ion‐blocking conditions. The resulting currents were measured and R_el_ was calculated as the slope of the voltage vs. current curve according to Ohm's law. Note that the contributions of the CC and the primer layer to the resistance were disregarded due to their insignificance compared to the electrode resistance.

#### 4.3.2 Ionic Conductivity Measurement

Symmetric electron‐blocking InLi/Sep/Cathode/Sep/LiIn cells were assembled to determine the ionic resistance R_ion_. 16 mm free‐standing cathodes without CC were compacted at 683 MPa for 5 min. 14 mm separators were compacted at 300 MPa for 5 min. One separator was placed on each side of the cathode and pressed with 525 MPa for 5 min. 12 mm InLi die‐cuts (prepared as in 4.4.1) serve as lithium reservoir on each side and were pressed onto the stack at 32 MPa for 2 min. Then, the cells were further assembled as in 4.3.3. Note that the contact areas between anodes and cathode with CC are covered by isolating tape to prevent a short‐circuit. Potentiostatic EIS measurements were recorded from 7 to 10 MHz with a 10 mV sinusoidal perturbation voltage. Based on the approach from Minnmann et al. and Siroma et al., resistance contributions of ion and electron conduction as well as interface resistances within the composite cathode are described via a T‐type (open‐open) transmission line model (TLM) [53, 78]. Non‐faradaic behavior was assumed for fully lithiated NCM, i.e., assuming no (de‐)intercalation processes. Values for R_ion_ were obtained by fitting via the RelaxIS 3 software (rhd Instruments, Germany). Kramers‐Kronig tests (determining the extent of linearity, causality, stability, and finiteness) were used to exclude high and low frequency noise. Thus, valid impedance data points are used for fitting.

#### 4.3.3 Pouch Cell Testing

InLi anodes were prepared by pressing In and Li at 73MPa for 10min in a uniaxial press (54KB1600M, Maassen, Germany). 14 mm die‐cuts of the InLi alloy were used as anodes. The cathode is 12 mm in diameter and was compacted at 800 MPa for 5 min. The separator is 16 mm and was pressed at 300 MPa for 5 min. Then, the cathode was centered on the separator and pressed with 6 t for 5 min. The anode was also concentrically placed on the other side of the separator and pressed with 0.5 t for 1 min. The cell stack was connected to CC tabs (Cu‐Ni on the anode, Al‐Al on the cathode) and vacuum sealed (C70, Multivac, Germany) at an absolute pressure of 50 mbar within a pouch bag. The cells were pressurized by four springs to 12 MPa.

The cells were tested by a potentiostat (VMP‐300, BioLogic, France) at 25°C.

Potentiostatic EIS measurements of the cathode half cells were conducted as in 4.3.2. The equivalent circuit model is based on the approach from Ruess et al. [6] and Mendizabal et al. [70]. Fitting was performed as in 4.3.2. DRT analysis was conducted in RelaxIS 3 software (rhd Instruments, Germany) with default settings: 1st derivative, Gaussian function, regularization parameter λ^*^ = 0.01, and shape factor 0.5.

For cell cycling, the upper and lower cut‐off voltages are 2.7 and 4.2 V vs. Li^+^/Li, respectively. Notably, for the InLi alloy anode, a stable potential of 0.62 V vs. Li^+^/Li was reported [79]. For formation, the cells were charged and discharged with 0.1 C constant current for two times. The potentiostatic charge test was performed from a discharged state with a chronoamperometry charge step at 4.2 V vs. Li^+^/Li. The stop criteria are 8 h time and a minimum current of 100 µA (∼ 0.03C). Then, for the standard galvanostatic charge rate test, the charge rates are 0.1 C, 0.5 C, and 1 C with constant current constant voltage (CCCV) discharge at 0.1 C.

For the calculation of the charge capacity *Q*_specific,charge_ at a given current *I* over time *t* of the chronoamperometry potentiostatic charge test, the following equation was used:

(4) $$Q_{\mathrm{specific},\mathrm{charge}}\left(t\right)=\frac{Q\left(t\right)}{m_{CAM}}=\frac{\int_{0}^{t}I\left(t\right)dt}{m_{CAM}}\;\left[mAh{g^{-1}}_{NCM}\right]$$

Based on the electrode weight (without current collector) and the CAM weight fraction, the CAM mass *m*_CAM_ was derived. Then, the C‐rate was determined from the calculated cell capacity *Q*_cell,theoretical_ (Equation S4) as follows:

(5) $$C-rate\left(t\right)=\frac{I\left(t\right)}{Q_{cellt,theoretical}}\;\;\;\;\left[h^{-1}\right]$$

Chronoamperometry data fitting was performed with the *Nonlinear Curve Fit Tool* from *OriginLab* (2019) and the fit parameters *Q*_0_, τ, and *n*. The following fit function was used:

(6) $$Q_{specific,charge}\left(t\right)=Q_{0}\;\left[-{\left(\tau \;C-rate\left(t\right)\right)}^{n}\right]\;\left[mAh{g^{-1}}_{NCM}\right]$$

The fitting metrics are summarized in Table 3.

**TABLE 3:** Fit parameters and error for chronoamperometry measurements.

Cell configuration	Q*_0_* ^a^ [mAh g^−1^_NCM_]	*τ* [h]	*n* [‐]	R^2^ [‐]
Extruder	190	1.732 ± 0.001	1.355 ± 0.001	0.997
Batch	190	3.283 ± 0.001	1.225 ± 0.001	0.996

^a^ Parameter fixated after initial fit iterations.

#### 4.3.4 Scanning Electron Microscopy, Energy Dispersive X‐Ray Spectroscopy

SEM images were recorded with an acceleration voltage of 5 kV, a beam current of 200 pA, and 8.5 mm working distance (Gemini 450, Zeiss, Germany). In addition, samples were analyzed via EDX to determine their elemental composition and distribution. To improve the signal intensity, the voltage and current were increased to 20 kV and 1 nA, respectively.

#### 4.3.5 X‐Ray Photoelectron Spectroscopy

For XPS measurements, samples were mounted conductively by either pressing powders into dents or glueing electrodes using copper tape onto a stainless‐steel stub in an argon‐filled glovebox. Samples were then transferred without exposure to atmosphere in a custom‐made inert transfer holder (Kratos, UK) into the XPS (Axis Supra, Kratos, UK). Data collection was performed at a pressure p ≤ 10^−8^ torr using a monochromatized Al K_α_ source (1486.6 eV) and active charge neutralization with a filament current of 0.45 A, a filament bias of 1 V and a charge balance at 5 V. For surface‐charge neutralization, C 1s spectra were recorded for each sample in the binding energy (BE) of 305–272 eV, with a step size of 0.1 eV, 400 ms dwell time and 2 sweeps. The S 2p core level was collected in the range of 175–150 eV, in 0.2 eV increments, 1500 s dwell time and a total of 3 sweeps. The low BE region was collected from 85 to 45 eV, in 0.1 eV steps, 800 ms dwell time and 3 sweeps. In addition to that, survey scans, Cl 2p, P 2p, and Ni 2p were collected (data not shown). After spectra collection, the BE was corrected using the adventitious carbon signal at 284.8 eV. Fitting was performed in CasaXPS (Version 2.3.24, UK) using a Gaussian‐Lorentzian fit function (GL(30), 30 % Lorentzian) after subtraction of a Shirley background [80].

Quantifying layer thicknesses of a species at the surface was calculated based on the work of Alexander et al. with λ = 3.5 nm using Beer–Lambert law [81].

#### 4.3.6 X‐Ray Diffraction

X‐ray diffractograms have been recorded from powdered samples sealed in Hilgenberg capillaries (0.5 mm) in Debye‐Scherrer geometry on a Bruker D8 Advance and using the wavelengths of copper K_α1,2_ radiation as radiation source. The samples were measured using a Goebel mirror for beam parallelization, a divergence slit of 0.6 mm, a receiving slit of 5 mm, primary and secondary Soller slits of 2.5 °. The samples were measured in the range from 10 to 120 ° 2θ in steps of 0.001 ° with 500 ms per step.

By using TOPAS [82].⁠ Rietveld refinements and quantitative phase analysis [83, 84, 85]⁠ have been performed in order to evaluate the phase content of the mixtures. The instrumental broadening has been corrected by determining the instrumental resolution function using powdered silicon (NIST SRM 640 g [86]⁠, a  =  0.5431109 nm) sealed in a capillary as an external reference material. The pure pristine materials were measured separately and the phase content of NCM in the mixtures were kept constant at 82.4 wt.%. The conductive carbon C65 was modelled as partial or not known crystal structure (PONKCS) [87] using space group Pmmm with lattice parameters *a*  =  42.48 Å and *b*  =  *c*  =  0.1 Å, whereas for NCM [88]⁠ and Li_6_PS_5_Cl [89]⁠ the respective reported crystal structures have been used as starting points for the refinements. The amorphous content was estimated assuming a density of 1.6 g cm^−3^ for C65. The total occupational factors of the metal sides in NCM have been kept constant to the values obtained from ICP‐OES. In the refinement of the pure pristine NCM the displacement parameters for the Wyckoff *3a* sites were set to a reasonable value, whereas these were refined for the *3b* and *6c* sites. For the subsequent quantitative phase analyses the displacement parameters were then kept constant. According to the low fractions of the other components in the phase mixtures, the crystallite size (i.e., volume weighted mean column length) and microstrain broadening have only been refined for NCM throughout the whole series.

#### 4.3.7 Laser Diffraction

Particle size distributions were measured using a particle size analyzer (Anton Paar 1190, Germany) via laser diffraction in p‐xylene with ultrasonic dispersion. Each material was measured six times on the same sample.

#### 4.3.8 Tensile Strength Test

The tensile strength of electrode samples was quantified using a pull‐off test setup under dry room atmosphere at room temperature. The measurements were performed by a ZwickRoell UPM Z020 (Germany) universal testing machine equipped with a 500 N load cell and round adhesive stamps (diameter 16 mm). 16 mm die‐cuts were punched from dry‐coated electrode sheets (without compaction by uniaxial press) and fixated between upper and lower stamps using adhesive tape. A pre‐pressing step of 500 N for 30 s was applied to promote uniform contact at the interface between electrode specimen and adhesive tape. Then the stamps were connected to the clamps in the testing machine. The tensile load was applied perpendicular to the coating plane (normal direction, 0°) with a speed of 100 mm min^−1^ until failure, while force–displacement data were recorded continuously. The tensile strength of electrodes was calculated by normalizing the peak force at failure to the sample area and reported in N cm^−2^. Three samples per electrode configuration were measured. Failure modes were classified as adhesive (interface) or cohesive (within the coating).

## Author Contributions

**Michael Wolf**: conceptualization, investigation, writing – original draft, methodology, validation, visualization, formal analysis, data curation. **Aaron Stoeckle**: investigation. **Milad Hosseini**: data curation. **Johannes Kriegler**: writing – review and editing. **Korbinian Huber**: writing – review and editing. **Rebecca Wilhelm**: methodology, data curation. **Maximilian A. Plass**: methodology, data curation. **Simon Lux**: writing – review and editing, supervision.

## Conflicts of Interest

The authors declare no conflicts of interest.

## Supporting information




**Supporting File**: advs78248‐sup‐0001‐SuppMat.docx.

## Data Availability

The data that support the findings of this study are available from the corresponding author upon reasonable request.
