# Supplementary material for: Microstructure Evolution in Dry‑Coated Sulfidic All‐Solid‐State Battery Cathodes: Investigating Core–Shell Particle Morphology
Source: Adv Sci (Weinh). 2026 Oct 12:e78248. Online ahead of print. doi: 10.1002/advs.78248 (PMC13658891; doi:10.1002/advs.78248)
Supplement: Supplementary file 1 [file ADVS-9999-e78248-s001.docx]

Article Highlights:

- Systematic comparison of continuous extruder mixing vs high‑shear batch mixing for dry‑coated sulfidic ASSB cathodes.
- Scalable dry coating via direct calendering in dry room atmosphere.
- Cathode composition: high-Ni NCM85 (D_50_ 3.8µm, 82 wt% CAM), LPSCl solid electrolyte, PTFE binder (0.5 wt%); electrode areal capacity 3.2 mAh cm^-2^.
- Microstructure analysis conducted with SEM/EDX, XRD, XPS, and particle size measurements.
- Extruder mixing achieves enhanced PTFE binder fibrillation and mechanofusion CAM–SE core–shell coating for small particle system compared to batch mixing.
- Cells with extruder mixed electrodes show enhanced CAM utilization and rate capability due to more efficient Li-ion transport.
